# Role of an Online Skill-Based Mindfulness Program for Healthcare Worker's Resiliency During the COVID-19 Pandemic: A Mixed-Method Study

**DOI:** 10.3389/fpubh.2022.907528

**Published:** 2022-07-14

**Authors:** Soyeon Kim, Jennifer Crawford, Sarah Hunter

**Affiliations:** ^1^Waypoint Research Institute, Waypoint Centre for Mental Health Care, Penetanguishene, ON, Canada; ^2^Department of Psychiatry and Behavioural Neurosciences, McMaster University, Hamilton, ON, Canada; ^3^Faculty of Health Sciences, Ontario Tech University, Oshawa, ON, Canada; ^4^Research and Innovation, Georgian College of Applied Arts and Technology, Barrie, ON, Canada

**Keywords:** mindfulness, COVID-19, healthcare workers, resiliency, online delivery

## Abstract

The COVID-19 pandemic has highlighted the mental health care needs of health care workers. The primary aim of this study was to explore the effects of an online mindfulness program on resiliency in health care workers during the pandemic using a mixed-methods approach. An online 4-week mindfulness program was delivered to healthcare workers (*N* = 130) in Ontario, Canada. Resiliency was assessed at 3-time points (i.e., pre, post, and 1-month follow-up), and the mindfulness program's efficacy on resiliency was analyzed using linear regression. Semi-structured interviews (*N* = 10) were conducted to substantiate their experiences. Healthcare workers' resiliency significantly increased after the mindfulness program compared to the baseline, maintaining the effect after 1 month in both unadjusted and adjusted regression models. These findings were further bolstered by the positive experiences shared by participants highlighting the program's efficacy on empathy and resiliency. Evidence suggests that mindfulness is associated with promoting resiliency in healthcare workers and may be an important strategy to promote resiliency in this population.

## Introduction

COVID-19 is one of the most traumatic events in the 21st century, especially for healthcare workers worldwide (1, 2). 1 in 5 healthcare workers reported experiencing depression and anxiety-related symptoms (3). Due to long hours and increased workload, healthcare workers are experiencing psychological stressors such as anxiety, depression, and burnout that have reduced their quality of life (4). Further, lived-experience narratives of healthcare workers' well-being during the first wave of the pandemic reveal that most of the healthcare workers experienced worsening mental health well-being due to stigma, fear, guilt and isolation (5).

In the province of Ontario, Canada, the first case was reported on January 25, 2020, and community transmission was documented on March 1, 2020, in British Columbia (6). Within days of this announcement, the Ontario provincial government declared a state of emergency. This declaration limited the activities and movement of individuals to reduce contact and the spread of the virus (7, 31). This included the government-mandated closure of non-essential businesses, all indoor recreational programs, schools, public libraries, theaters, and some days later, all outdoor recreational spaces, including parks and walking trails. Public gatherings were also limited to < 50 people. While some of these restrictions were lifted by June 22, 2020, the provincial state of emergency continued until July 24, 2020. Over the next 2 years, a provincial state of emergency has been redeclared on different occasions, and ongoing changes to these restrictions have continued in an effort to slow transmission and alleviate pressures on the healthcare system. Research has revealed that the constant demands and pressures of the COVID-19 pandemic have significantly affected healthcare workers' mental health and well-being (8).

The psychological stressors during the pandemic compromise healthcare workers' capacity to provide care, thus endangering the stability and quality of patient care (9) highlight the importance of acceptance-based coping in their recent perspective article on recovery from COVID-19. The authors indicate that fully accepting emotions arising to the mind is critical to achieving a longer-term resiliency. Resiliency, defined as “the ability of adults… to maintain relatively stable, healthy levels of psychological and physical functioning… as well as the capacity for generative experiences and positive emotions (pp. 20-21)”(10), is vital for healthcare workers and society (1, 9, 11–13). Thus, implementing robust health and educational services that promote resiliency for healthcare workers is paramount to supporting recovery from the global epidemic (1, 14, 15).

Mindfulness programs embody awareness and acceptance-based practices and promote connectedness that can be used to build resiliency (9, 16). Mindfulness is defined as a practice that involves focusing awareness on the present moment while acknowledging and accepting one's feelings, thoughts, and bodily sensations without judgment (17). With over two decades of mounting scientific research demonstrating benefits—from fortifying the immune system to reducing stress and anxiety and improving overall well-being—mindfulness-based intervention has been used to mitigate emotional challenges (18–20). Mindfulness programs are a promising tool supporting resiliency for healthcare workers (9, 16). A recent systematic review on the psychological effects of online-based mindfulness programs during the pandemic that included 6 randomized controlled trials concluded that the online mindfulness program could be useful for reducing anxiety, depression and stress levels of the general population, healthcare workers and patients (32).

However, to our knowledge, whether it can effectively enhance resiliency among healthcare workers during COVID-19 has not been studied yet. There is a growing interest in creating accessible strategies for healthcare workers to promote better mental health and resiliency. The outcome of this study, grounded both on quantitative and qualitative findings, responds to the critical need to cultivate a resilient society amid a global pandemic. Lessons learned will guide healthcare services for healthcare workers (e.g., well-ness programming) throughout physical distancing and beyond as the pandemic slowly transitions to an endemic status.

## Methods

### Study Design and Participants

A mixed-methods approach was used to examine the role of the 4-week online Mindfulness Ambassador Program (MAP; developed by a non-profit organization, Mindfulness Without Borders), in fostering resiliency during the pandemic and exploring the participant's and facilitator's experience. A total of 130 healthcare workers in Ontario, Canada, participated in the study through a frontline well-ness program at a psychiatric hospital and completed pre-, post-, and follow-up surveys (mean age 31–50 = 53.8%; female = 93%). For the qualitative analysis, we predicted that basic themes may emerge after 4–6 interviews and that saturation will signal the end of recruitment. Among 130 healthcare workers, we aimed to recruit ten random participants for semi-structured interviews through multiple follow-up emails. As saturation began to emerge after seven interviews, where no new data or themes emerged, and as a result, no new codes, we stopped the recruitment for interviews. The interviewees were all women. 67% of the women were between the ages of 31 to 50 years old. 45% had a non-clinical role (e.g., administration, research, IT), and another 45% provided direct patient care. 43% of the interviewees had more than 20 years of working experience in a healthcare setting, and another 43% had l <11 years of experience.

### Procedure

The 4-week online Mindfulness Ambassador Program (MAP) was offered to healthcare workers *via* zoom through the institutions' frontline wellness program (October 2020 to March 2021). Any active health care worker (regardless of their history of psychiatric illness or substance use) in the province of Ontario seeking mental-wellness assistance during the COVID-19 pandemic could self-refer and register for the 4-week online mindfulness program through the Frontline Well-ness Program. Registering in the mindfulness program did not constitute registration in this research study. Those who are no longer in active employment as health care workers were excluded from participating in the study but still had access to the MAP if they chose. All health care workers who enrolled in the 4-week MAP were sent a participant information letter by the MAP facilitators, inviting them to participate in the study. After obtaining consent, a pre-survey was administered before the program began.

The MAP program was retooled to provide healthcare workers and the community an in-time well-being service in response to the pandemic. Retooling the MAP program morphed a face-to-face, 12- week and 12-h program into a virtual, 4- week, 2-h skill-based course. After obtaining consent, a pre-survey was administered before the program began. Each mindfulness session ran once a week for 30 min, with one certified MAP facilitator assigned to each group (max 20). A post-survey was administered immediately after the fourth session, with a follow-up survey 1 month after the last session. After the post and follow-up survey, participants were offered a $20 gift card as a thank-you gift for their time completing the surveys. Surveys for this quantitative component were conducted and completed online. Followed by the post-surveys, the research team conducted semi-structured interviews with ten randomly selected participants, and program facilitators from April through mid-May 2021 *via* a secure online platform (i.e., Zoom). The interviews took an hour, and an additional $25 gift card was offered. All participants provided consent to audio-record the interviews. All recorded data and transcriptions were stored in a password-protected laptop.

### Analysis

#### Quantitative

Linear regression models using the generalized least squares (GLS) approach, allowing more efficient estimates and unbiased regression parameters for panel data, were conducted to evaluate the efficacy of the mindfulness program on healthcare workers' resiliency, measured by Nicholson McBride Resilience Questionnaire (NMRQ). NMRQ is a 12-item survey with Likert-type responses designed to measure resilience, which is defined as one's capacity to bounce back from extreme occasions or triumph in the face of hardship (Clarke, 2010). Scores range from 12 to 60, where higher numbers are indicative of greater resilience. First, an unadjusted regression model was conducted to examine the change in resiliency total score over time (baseline, post-intervention, and 1-month follow-up after completing the mindfulness program). Next, participant characteristics such as age, sex, healthcare worker's role (clinical, non-clinical), work location (front line or virtual/online), years of occupational service, and mindfulness related variables such as previous exposure to mindfulness program (Yes/No), the number of sessions attended, and the frequency of mindfulness practice was added to the model (Adjusted model). Coefficients and 95% confidence intervals (CIs) are reported. Software for Statistics and Data Science (STATA; V.16.0) was used.

#### Qualitative

The interview protocol was developed during a research advisory meeting with seasoned mindfulness practitioners and the research team. All transcriptions were member-checked by participants to ensure the interview data provided an interpretation that resonated with the participant. Thematic analysis (TA), a method to systematically identify, organize and offer insight into patterns of meaning (themes) across a dataset, was used (21) to identify common themes that arose among facilitators' and participants' experiences with 4-week online mindfulness programs and the cultivation of resiliency. The iterative stages of reviewing, defining, and naming themes were completed using the entire dataset. All qualitative analyses were conducted using NVivo software.

## Results

Fifty percent of participants reported working in healthcare for < 10 years, 53% reported working directly with patients, and 64% of participants had no previous mindfulness experience. Participants reported practicing mindfulness 1–3 times per week in both the post and follow-up surveys (post, 65%; the follow-up, 69%). Table 1 presents the resiliency score based on the participant characteristics and mindfulness-related variables at three-time points. Table 2 presents the unadjusted and adjusted regression model findings. Based on the unadjusted model, healthcare workers' resiliency significantly increased after the mindfulness program compared to the baseline, maintaining the effect after 1 month. The efficacy of the mindfulness program was sustained after adjusting for participant characteristics and mindfulness-related correlates. Older age (compared to < 30 years old) and the number of mindfulness practices (4–7 times a week) significantly contributed to resiliency.

**Table 1:** Resiliency score for three-time points based on participant characteristics and mindfulness related variables (mean, SD).

	**Baseline**	**Post-intervention**	**1-month follow up**
**Age**			
<31	39.15 (5.33)	38.57 (6.55)	41.55 (6.41)
31–50	40.48 (5.55)	43.32 (6.61)	42.97 (6.42)
>50	43.60 (6.33)	41.88 (6.75)	44.23 (5.62)
**Years worked**			
<11	41.56 (5.18)	43.09 (7.11)	43.89 (4.99)
11–20	42.44 (3.48)	42.89 (4.05)	44.40 (4.62)
>20	42.71 (6.94)	42.00 (7.50)	43.50 (5.78)
**Mode of care**			
Direct	41.80 (5.72)	42.50 (6.55)	43.30 (5.41)
Indirect	40.24 (6.09)	41.11 (7.30)	42.40 (6.92)
**Mindfulness exposure**			
No	41.25 (6.17)	42.19 (6.96)	42.56 (6.82)
Yes	41.07 (5.59)	41.70 (6.63)	43.83 (4.73)
**# of mindfulness practice**			
Never	40.00 (6.93)	40.00 (8.93)	39.25 (8.10)
1–3 times/week	40.39 (6.43)	41.42 (6.62)	43.03 (6.43)
4–7 times/week	41.82 (6.31)	44.53 (5.56)	45.08 (4.98)
**# MAP sessions attended**			
1–2 sessions	39.82 (8.38)	39.33 (8.92)	41.10 (8.95)
3 sessions	40.71 (7.05)	42.33 (6.44)	45.00 (5.93)
4 sessions	41.10 (5.27)	43.39 (5.02)	43.17 (5.34)

**Table 2:** Associations between resiliency and participant characteristics and mindfulness related variables (Coefficients, 95% CI).

	**Unadjusted model**	**Adjusted model**
**Time**		
Baseline	Ref	Ref
Post–intervention	1.09 (0.12–2.05)*	1.26 (0.18–2.34)*
Follow up	1.66 (0.78–2.54)***	1.73 (0.58–2.87)**
**Age**		
<30		Ref
31–50		5.07 (1.69–8.45)**
>51		4.93 (0.55–9.31)*
**Years worked**		
<11		Ref
11–20		−1.18 (−4.53–2.17)
>20		−1.53 (−4.49–1.44)
**Mode of care**		
Direct		Ref
Indirect		0.86 (−2.95–4.66)
**Mindfulness exposure**		
No		Ref
Yes		−2.53 (−5.45–0.38)
**# of mindfulness practice**		
Never		Ref
1–3 times/week		4.93 (−0.60–10.45)
4–7 times/week		8.56 (3.28–13.84)**
**# MAP sessions attended**		
1–2 sessions		Ref
3 sessions		0.67 (−3.59–4.94)
4 sessions		−0.69 (−3.89–2.51)

* < 0.05, ** < 0.01, *** < 0.001.

Participants' qualitative narrative substantiated and complemented the quantitative findings by providing a nuanced account based on their experience. In general, the positive experiences shared situated the program's positive effect on empathy and resiliency by providing time and space for them to practice self-care and teaching strategies that helped them embody mindfulness. Participants shared that the 4-week program helped them flip the script and develop a better understanding of the importance of self-care and self-compassion, “*It solidified the fact that you know, I am worth it that I need to focus on taking care of myself rather than always focused on taking care of others (31-50 yrs old, female, direct patient care*,<*11 years of healthcare work experience)*.” For this participant and others, the availability of the program and the program itself encouraged them to prioritize their own self-care, provided an “*important reminder*” or the space to actualize self-care. Some also emphasized that the program helped them establish a routine and dedicate time to practice. Others shared that they found the time and space provided with the program a welcome reprieve from the complexity of the support and care they were providing others in their roles. Others who echoed this sentiment shared the responsiveness of the program to provide just in time support, “*I'm glad that I was able to participate, and it was just at the right time where I kind of felt like I finally got into a bit of a crisis myself...It was very helpful that way and I just really appreciated it was there. Sort of when I needed it (31-50 yrs old, female, virtual patient care*,<*11 years of healthcare work experience)*.”

Some participants shared that they were applying the strategies taught in the program and that these strategies were supporting their well-being. Others admitted that they were not applying the practice strategies. However, participants of this study all shared how they were embodying mindfulness, revealing that the effects of the strategies taught had become habitual or part of participants' resiliency toolkit. Some shared that they were pausing to take deep breaths during moments of heightened anxiety or frustration or seizing 5 min to ground by sitting in the car, awakening awareness, beginning to reveal the many ways in which the practices taught were being integrated: “*Because it (the body scan) just made me relax better, and I sometimes still do that when I come home from work, when I told you that I feel like I'm still kind of in overdrive. I try to just go from the top of my head to the bottom of my toes and just sort of and I could do that quietly without even if my husbands in the room, you can just sort of inwardly focus on yourself (51-65 yrs old, female, non-clinical support).”*

## Discussion

Providing accessible and flexible resources to improve resiliency in healthcare workers is critical during the pandemic (2, 9, 15). This study investigated the efficacy of a 4-week online mindfulness program and contributing factors in resiliency among healthcare workers during the COVID-19 pandemic using a mixed-method approach. Resiliency significantly increased after the 4-week online mindfulness program, and the effects were maintained after 1 month. Practicing mindfulness techniques frequently (4–7 times a week) and age over 30 years significantly and positivelyassociated with resiliency. The qualitative narratives revealed that the connection between the positive framing of the mindfulness program as self-care conceptualized why participants were drawn to attending, and their shared challenges with carving out time for practice was balanced with the many ways in which practice is being integrated into daily living, something that may not have been captured in reports of frequency of practice.

The results of this study support the efficacy of a 4-week online mindfulness program enhancing resiliency in healthcare workers during the pandemic. The positive impact of mindfulness practice was attributed to the number of daily mindfulness practices and being able to integrate practices into personal and professional lives, reaffirming the importance of pursuing efforts to support continued practice. Our findings on resiliency uniquely add to the growing literature on the efficacy of mindfulness practice on mental well-being during the pandemic (1, 22–24). For instance, mindfulness practice to awaken present moment awareness and practice non-judgmental awareness can lead to positive mental health outcomes during the pandemic, such as improved empathy, self-compassion among healthcare professionals (22), pandemic-related distress (23–25) and emotional problems such as anxiety and depression (24, 25) during COVID-19.

Our study is limited by the small number of interviews to reach data saturation, as a minimum of 13 cases is recommended (21). Despite the low number of interviews, we believe that the resultant response rate provided “rich” insight into the emic experience of healthcare workers. Whereby thick data refers to a large quantity of data, rich refers to its quality. This study's data was layered, nuanced and enriched the understanding of an experience or phenomenon (26). Further, most healthcare workers who participated in the study are mostly located in rural areas of the province, and mostly females limiting the ability to generalize the findings. Women are the dominant gender group among healthcare workers in Canada. A recent scoping review (27) indicates that female healthcare workers show high anxiety, depression, and burnout during the pandemic. Despite the skewed sex ratio and region, we believe our study provided the groundwork for mitigating female healthcare workers' mental well-being in rural regions during the pandemic. Additional research using a randomized controlled trial design and comparing the efficacy of mindfulness programs with a different mode, timing, and frequency on resiliency with diverse healthcare workers in broader area is warranted to further determine the efficacy of mindfulness programs in healthcare workers' resiliency.

Despite the limitations, to our knowledge, this is the first study that explored the role of an online mindfulness program to enhance resiliency in healthcare workers, bolstering the online delivery of mindfulness programs during the pandemic. Furthermore, we used panel data and conducted linear regression analysis using the generalized least squares (GLS) approach for efficient estimation and unbiased regression parameters to evaluate the efficacy of the mindfulness program. Knowledge gained from this study will guide organizational development initiatives seeking to build resiliency using mindfulness-based intervention programs. It is worth noting that while mindfulness has been associated with positive health outcomes in this population, research has found barriers to engaging in mindfulness practices that must be considered (28–30). For instance, Valley et al. reported internal barriers to health care workers' adopting mindfulness-based practices include high levels of stress, difficulties with attention, and a lack of prioritization for self-care activities within the workplace. These findings have likely only been further exacerbated by the demands of the pandemic. Further research is warranted to explore how to best tailor mindfulness-based programs to improve adherence to future interventions and the long-term adoption of these practices. Given these findings, at an organizational level, protected time to participate in wellness activities (e.g., mindfulness practice) should be explored as a needed and essential part of preventing mental health and building resilience within health care workers.

In conclusion, our findings have important public health implications, as protecting and restoring healthcare workers' health and well-being is of immediate concern to public health professionals during the COVID-19 pandemic. These important considerations unearthed through this study may help guide pedagogical and instructional changes in mindfulness-based intervention programs designed to facilitate resiliency in the future. The evidence-base produced through this study will support the widespread implementation of an online mindfulness-based intervention, delivered throughout the period of physical distancing and beyond, as we slowly transition to a “new normal.”

## Data Availability Statement

The data that support the findings of this study are available on request from the corresponding author [SK]. The data are not publicly available due to them containing information that could compromise research participant privacy/consent.

## Ethics Statement

The studies involving human participants were reviewed and approved by the Institution's Ethics Review Board (Waypoint Center for Mental Health Care, Protocol ref. # HPRA#20.07.27). The patients/participants provided their written informed consent to participate in this study.

## Author Contributions

SK conducted the quantitative data analysis and drafted the manuscript. SH conducted semi-structured interviews and qualitative data analysis. All authors contributed to the conceptualization of this study. All authors contributed to drafting the manuscript, provided critical revisions, and approved the final manuscript.

## Funding

This study was supported by a Partnership Engagement Grant funded by the Social Sciences and Humanities Research Council of Canada (Award # 1008-2020-1033) and the TD Ready Commitment Grant (Better Health—Innovative Solutions).

## Conflict of Interest

SH worked as a facilitator with Mindfulness Without Border (MWB) during the data collection period facilitating mindfulness programs for other institutions (not related to this study) and was no longer in contract with MWB during the manuscript preparation period. The remaining authors declare that the research was conducted in the absence of any commercial or financial relationships that could be construed as a potential conflict of interest.

## Publisher's Note

All claims expressed in this article are solely those of the authors and do not necessarily represent those of their affiliated organizations, or those of the publisher, the editors and the reviewers. Any product that may be evaluated in this article, or claim that may be made by its manufacturer, is not guaranteed or endorsed by the publisher.

## References

[1] Heath C Sommerfield A von Ungern-Sternberg BS. Resilience strategies to manage psychological distress among healthcare workers during the COVID-19 pandemic: a narrative review. Anaesthesia. (2020) 75:1364–71. 10.1111/anae.15180 32534465 PMC7323405

[2] Rillera Marzo R Villanueva Iii EQ Chandra U Htay MNN Shrestha R Shrestha S. Risk perception, mental health impacts and coping strategies during COVID-19 pandemic among Filipino healthcare workers. J Public Health Res. (2021) 10(s2):jphr.2021.2604. 10.4081/jphr.2021.2604 34911287 PMC9131489

[3] Pappa S Ntella V Giannakas T Giannakoulis VG Papoutsi E Katsaounou P. Prevalence of depression, anxiety, and insomnia among healthcare workers during the COVID-19 pandemic: a systematic review and meta-analysis. Brain Behav Immun. (2020) 88:901–7. 10.1016/j.bbi.2020.05.026 32437915 PMC7206431

[4] Kock DE Latham JH Leslie HA Grindle SJ Munoz M. A rapid review of the impact of COVID-19 on the mental health of healthcare workers: implications for supporting psychological well-being. BMC Public Health. (2021) 21:1–18 10.1186/s12889-020-10070-3 33422039 PMC7794640

[5] Creese J Byrne JP Conway E Barrett E Prihodova L Humphries N. “We all really need to just take a breath”: composite narratives of hospital doctors' well-being during the COVID-19 pandemic. Int J Environ Res Public Health. (2021) 18:2051 10.3390/ijerph18042051 33669828 PMC7921910

[6] Berry I. O'Neill M. Sturrock S. L. Wright J. E. Acharya K. Brankston G.. (2021). A sub-national real-time epidemiological and vaccination database for the COVID-19 pandemic in Canada. Sci. Data. 8:1–10. 10.1038/s41597-021-00955-2 34267221 PMC8282612

[7] Detsky AS Bogoch II. COVID-19 in Canada: experience and response. JAMA. (2020) 324:743–4. 10.1001/jama.2020.14033 32790824

[8] Maunder RG Heeney ND Hunter JJ Strudwick G Jeffs LP Ginty L. Trends in burnout and psychological distress in hospital staff over 12 months of the COVID-19 pandemic: a prospective longitudinal survey. J Occup Med Toxicol. (2022) 17:1–11. 10.1186/s12995-022-00352-4 35614505 PMC9132565

[9] Polizzi C Lynn SJ Perry A. Stress and coping in the time of covid-19: pathways to resilience and recovery. Clin Neuropsychiatry. (2020) 17:59–62. 10.36131/CN20200204 34908968 PMC8629051

[10] Bonanno GA. Loss, trauma, and human resilience: have we underestimated the human capacity to thrive after extremely aversive events? Am Psychol. (2004) 59:20. 10.1037/0003-066X.59.1.20 14736317

[11] Heymann DL Shindo N. COVID-19: what is next for public health? Lancet. (2020) 395:542–5. 10.1016/S0140-6736(20)30374-3 32061313 PMC7138015

[12] Shaw SC. Hopelessness, helplessness and resilience: The importance of safeguarding our trainees' mental wellbeing during the COVID-19 pandemic. Nurse Educ Pract. (2020) 44:102780. 10.1016/j.nepr.2020.102780 32272394 PMC7138173

[13] Smith GD Ng F Li WHC. COVID-19: Emerging compassion, courage and resilience in the face of misinformation and adversity. J Clin Nurs. (2020) 29:1425. 10.1111/jocn.15231 32155302 PMC7166833

[14] Djalante R Shaw R DeWit A. Building resilience against biological hazards and pandemics: COVID-19 and its implications for the Sendai Framework. Progress Disaster Sci. (2020) 6:100080 10.1016/j.pdisas.2020.100080 34171009 PMC7148717

[15] Serrão C Duarte I Castro L Teixeira A. Burnout and depression in portuguese healthcare workers during the covid-19 pandemic—the mediating role of psychological resilience. Int J Environ Res Public Health. (2021) 18:636. 10.3390/ijerph18020636 33451083 PMC7828555

[16] Behan C. The benefits of meditation and mindfulness practices during times of crisis such as Covid-19. Irish J Psychol Med. (2020) 37. 10.1017/ipm.2020.38 32406348 PMC7287297

[17] Kabat-Zinn J. Mindfulness-based interventions in context: past, present, and future. Clin Psychol: Sci Pract. (2003) 10:144–56. 10.1093/clipsy.bpg016

[18] Brady S O'Connor N Burgermeister D Hanson P. The impact of mindfulness meditation in promoting a culture of safety on an acute psychiatric unit. Perspect Psychiatr Care. (2012) 48:129–37. 10.1111/j.1744-6163.2011.00315.x 22724398

[19] Cohen-Katz J Wiley SD Capuano T Baker DM Shapiro S. The effects of mindfulness-based stress reduction on nurse stress and burnout, Part II: a quantitative and qualitative study. Holist Nurs Pract. (2005) 19:26–35. 10.1097/00004650-200501000-00008 15736727

[20] Ireland MJ Clough B Gill K Langan F O'Connor A Spencer L. A randomized controlled trial of mindfulness to reduce stress and burnout among intern medical practitioners. Med Teach. (2017) 39:409–14. 10.1080/0142159X.2017.1294749 28379084

[21] Clarke V Braun V. Thematic analysis *Encyclopedia of Critical Psychology*. Springer (2014). p. 1947–52.

[22] Pérula-de Torres LÁ Verdes-Montenegro-Atalaya JC Melús-Palazón E García-de Vinuesa L Valverde FJ Rodríguez LA. Comparison of the effectiveness of an abbreviated program versus a standard program in mindfulness, self-compassion and self-perceived empathy in tutors and resident intern specialists of family and community medicine and nursing in Spain. Int J Environ Res Public Health. (2021) 18:4340. 10.3390/ijerph18084340 33923868 PMC8073262

[23] Zhu JL Schülke R Vatansever D Xi D Yan J Zhao H. Mindfulness practice for protecting mental health during the COVID-19 pandemic. Transl Psychiatry. (2021) 11:1–11. 10.1038/s41398-021-01459-8 34050125 PMC8160402

[24] Antonova E Schlosser K Pandey R Kumari V. Coping with COVID-19: Mindfulness-based approaches for mitigating mental health crisis. Front Psychiatry. (2021) 12:322. 10.3389/fpsyt.2021.563417 33833695 PMC8021723

[25] Soklaridis S Lin E Lalani Y Rodak T Sockalingam S. Mental health interventions and supports during COVID-19 and other medical pandemics: a rapid systematic review of the evidence. Gen Hosp Psychiatry. (2020) 66:133–46. 10.1016/j.genhosppsych.2020.08.007 32858431 PMC7442905

[26] Dibley L. Analysing narrative data using McCormack's Lenses. Nurse Res. (2011) 18:c8458. 10.7748/nr2011.04.18.3.13.c8458 21560921

[27] Sriharan A Ratnapalan S Tricco AC Lupea D Ayala AP Pang H. Stress, burnout and depression in women in health care during COVID-19 pandemic: rapid scoping review. medRxiv. (2020). 10.1101/2020.07.13.20151183 34816168 PMC8594027

[28] Banerjee M Cavanagh K Strauss C. A qualitative study with healthcare staff exploring the facilitators and barriers to engaging in a self-help mindfulness-based intervention. Mindfulness. (2017) 8:1653–64. 10.1007/s12671-017-0740-z 29201248 PMC5693971

[29] Valley M Stallones L. A thematic analysis of health care workers' adoption of mindfulness practices. Workplace Health Safety. (2018) 66:538–44. 10.1177/2165079918771991 29806801

[30] Xu HG Tuckett A Kynoch K Eley R. A mobile mindfulness intervention for emergency department staff to improve stress and wellbeing: a qualitative study. Int Emerg Nurs. (2021) 58:101039. 10.1016/j.ienj.2021.101039 34333332

[31] Desson Z Weller E McMeekin P Ammi M. An analysis of the policy responses to the COVID-19 pandemic in France, Belgium, and Canada. Health Policy Technol. (2020) 9:430–46. 10.1016/j.hlpt.2020.09.002 33520640 PMC7832234

[32] Kim SD Yeun YR. Psychological effects of online-based mindfulness programs during the CoViD-19 pandemic: a systematic review of randomized controlled trials. Int J Environ Res Public Health. (2022) 19:1624 10.3390/ijerph19031624 35162646 PMC8835139

